# A hybrid approach of vision transformers and CNNs for detection of ulcerative colitis

**DOI:** 10.1038/s41598-024-75901-4

**Published:** 2024-10-21

**Authors:** Syed Abdullah Shah, Imran Taj, Syed Muhammad Usman, Syed Nehal Hassan Shah, Ali Shariq Imran, Shehzad Khalid

**Affiliations:** 1 Department of Creative Technologies, Faculty of Computing and Artificial Intelligence, Air University, Islamabad, 44000 Pakistan; 2 College of Interdisciplinary Studies, Zayed University, 144534, Abu Dhabi, United Arab Emirates; 3 Department of Computer Science, Bahria School of Engineering and Applied Sciences, Bahria University, Islamabad, 44000 Pakistan; 4 Department of Computer Science, Norwegian University of Science and Technology, Gjøvik, 2815 Norway; 5 Department of Computer Engineering, Bahria School of Engineering and Applied Sciences, Bahria University, Islamabad, 44000 Pakistan

**Keywords:** Computer science, Medical imaging

## Abstract

Ulcerative Colitis is an Inflammatory Bowel disease caused by a variety of factors that lead to a serious impact on the quality of life of the patients if left untreated. Due to complexities in the identification procedures of this disease, the treatment timeline and quality can be severely affected, leading to further consequences for the sufferer. The difficulties in identification are due to high patients to healthcare professionals ratio. Researchers have proposed variety of machine/deep learning methods for automated detection of ulcerative colitis, however, several challenges exists including class imbalance problem, comprehensive feature extraction and accurate classification. We propose a novel method for accurate detection of ulcerative colitis with augmentation techniques to overcome class imbalance issue, a comprehensive feature vector extraction using custom architecture of Vision Transformer (ViT) and accurate classification using customized Convolutional Neural Network (CNN). We used the TMC-UCM and LIMUC datasets in this research for training and testing of proposed method and achieved accuracy of 90% with AUC-ROC scores of 0.91, 0.81, 0.94, and 0.94 for the endoscopic classes of Mayo 0, Mayo 1, Mayo 2, and Mayo 3 respectively. We have compared the proposed method with existing state of the art methods and conclude that the proposed method outperforms the existing methods.

## Introduction

Ulcerative Colitis (UC) is an Inflammatory Bowel Disease (IBD) occurs in individuals due to a combination of genetic and environmental factors along with abnormal reactions from the immune system^1^. It is divided into multiple stages of progression, which are further grouped into scoring systems like Mayo Endoscopic Score (MES) and Ulcerative Colitis Endoscopic Index of Severity (UCEIS): benign, mild, moderate, and severe. At the later stage of progression, the disease severely impacts the quality of life to the extent that full-time medical care may be necessitated for treatment. The detection of this disease is done through a variety of tests like imaging, biopsy, blood tests, and stool tests. Many forms of detection are not the optimal choices for this purpose, e.g., CT-Scan’s non-invasiveness limits its capabilities for Ulcerative Colitis and might even promote tumor growth and cause irritation on the lesions while the results might be obscured by any presence of higher gas contents in the colon^2^. Endoscopy is generally considered the best possible way as it provides a visual input regarding the patient’s current situation and even histological testing can be carried out through the samples extracted in this process^3^.

The treatment of this disease is complicated by difficulties in the correct identification of its symptoms in affected regions, and therefore, inter-observer disagreements are common, which can lead to delays in the actual treatment^4^. Researchers proposed method by draw out disease activity from the samples from genes to detect inherited capability of disease^5^ or by using a combination of symp bio-scopic solutions estimating neutrophils detection to uncover the activity of the disease through the Histogrammical test^6,7^. It is done by gene testing, pooling from research driven sampling to identify important genomes with high coherent relations to the disease’s existence by deriving and predicting results from clinical testing and variables related to patient habits to detect activity and severity^8^. Endoscopic studies have also been proposed for disease detection through image classification on the basis of the MES or UCEIS classification system for UC to detect progression of disease and relapsing through standard monitoring instruments^4,9–14^, for innovation such as the camera capsule technology^15^.

**Fig. 1:**
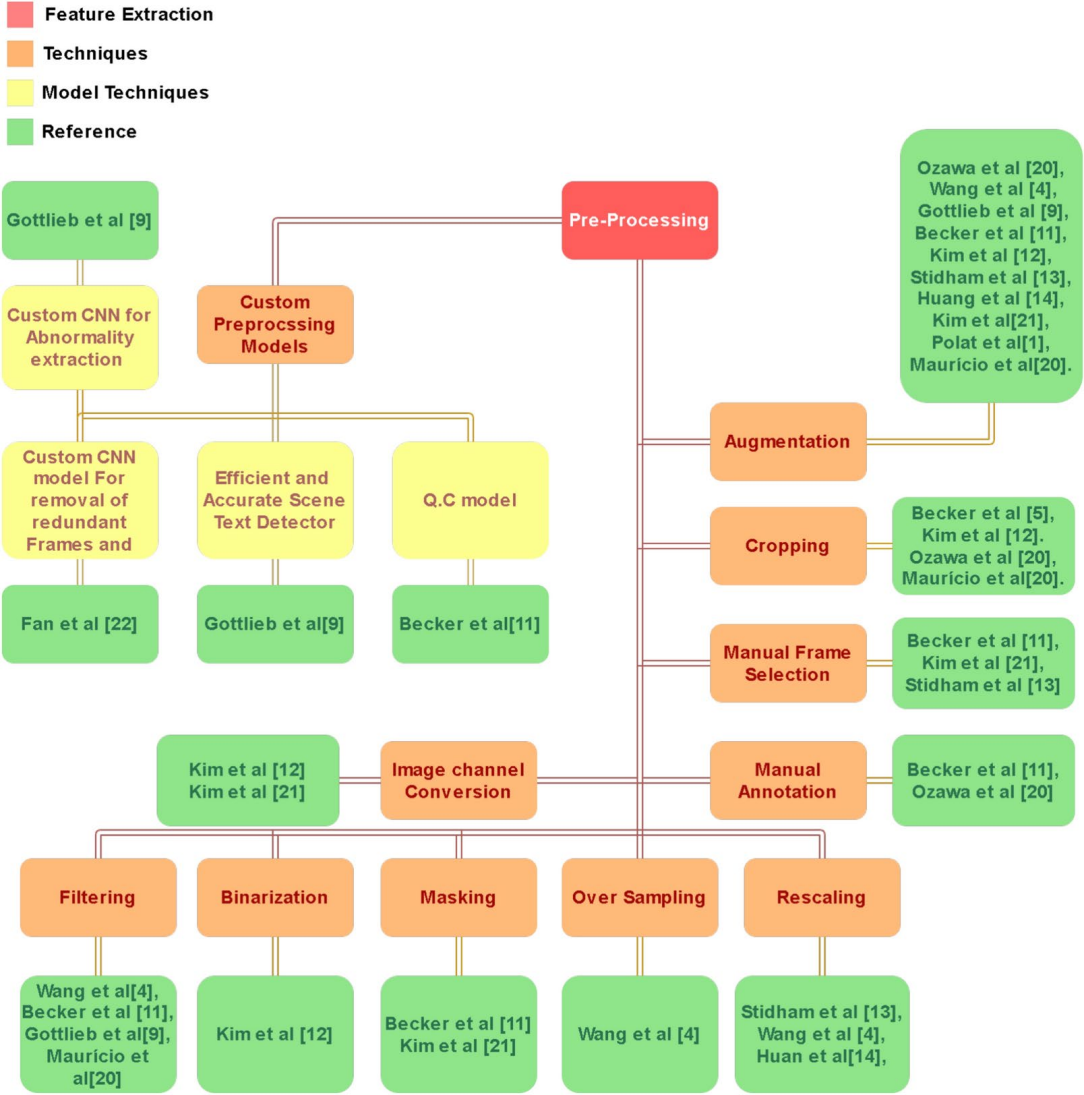
Preprocessing methods proposed by researchers in recent years.

Various symptoms of UC share similar symptoms with Crohn’s Disease (CD) with the observed difference among the diseases being the area of affliction, as declared by Davidson Principal and Practice of Medicine^3^, which is the colon or large intestine for UC and smaller intestine for CD. Several studies have been proposed that involve histological findings^16,17^ and genes sample for biomarker identification^18^. Similarly, our research led us to the classification of all 23 classes of IBD disease using endoscopic images by machine learning models, which not only classified the activity of UC but all the other labels of IBD in the HyperKavsir dataset^19^.

Despite significant progress in utilizing machine learning for Ulcerative Colitis detection, one of the key challenges that remain is addressing class imbalance within the datasets. Class imbalance can hinder the accuracy and reliability of machine learning models, particularly in identifying less frequent stages of the disease. The imbalance within datasets, such as HyperKavsir^19^, LIMUC^1^, and TMC-UCM^4^, often results in biased model predictions that favor the more populated classes. Therefore, addressing class imbalance is crucial to improving the model’s performance. Another challenge is comprehensive feature set extraction for accurate classification. Contributions of this research to the body of knowledge include:

- A novel hybrid model is proposed by combining a 3-fold Vision Transformer (ViT) and Convolutional Neural Network (CNN). By extracting features from the ViT and using averaging fusion with our dataset to enhance the CNN, the model demonstrates superior classification performance compared to using ViT alone.
- Implemented the High-Frequency Balancing and Augmentation technique to address class imbalance issues in Ulcerative Colitis datasets. This approach improved the model’s ability to handle uneven class distributions and enhances overall classification accuracy.
- Proposed hybrid model achieves high accuracies (up to 85% for ViT and 90% for CNN) and demonstrates strong AUC-ROC scores (0.91 for Mayo 0, 0.81 for Mayo 1, 0.94 for Mayo 2, and 0.94 for Mayo 3).
- The proposed model is designed to be continuously refined, improving efficiency and accuracy in disease identification and potentially alleviating the burden on medical professionals.

**Fig. 2:**
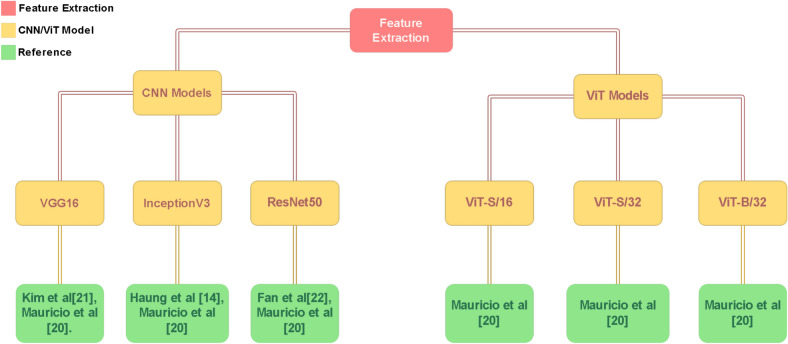
Feature extraction methods proposed by researchers in recent years.

## Related work

Experienced surgeons and gastroenterologists are required to identify different stages of Ulcerative Colitis using various methods such as biopsy, colonoscopy, and sigmoidoscopy. However, such experienced and qualified medical professionals are not available across all geographic regions of the world. Therefore, researchers have been trying to implement machine learning methods for automated UC detection and identification so that an easily scalable and resource-intensive solution can be designed to deal with this problem. Different research has been conducted on the basis of varying methodologies proposing unique ways of tackling these difficulties, and we, too, propose a different methodology that serves to widen the available strategies that can be utilized for further experimentation.

Figure 1 shows multiple techniques of preprocessing proposed by researchers for automated detection of Ulcerative Colitis. Cropping was utilized by Wang^4^, Becker^11^, Kim^12^ Maurício et al.^20^, and Ozawa^21^ while resizing was utilized in Wang^4^, Kim^12^ Stidham^13^, Huang^14^, Ozawa et al.^21^, Maurício et al.^20^ and Kim^22^. Other than Fan^23^, all of the rest applied augmentation, while Kim et al.^12^ used only normalization. Filtering was employed in Gottlieb et al.^9^ while manual frame selection was used in Becker et al.^11^, Polat et al.^1^, Ozawa et al.^21^, Maurício et al.^20^ and Kim^22^. Kim^12^ also chose grayscale conversion and binarization, while Becker^11^ used text removal and field of view masking for their preprocessing stage. Kim^22^ employed RGB to HSV conversion, Ozawa^21^ used annotation, Huang et al.^14^ used pixel value rescaling, and Kim^12^ used gray scaling. Gottlieb et al.^9^ used abnormality extraction and sequential processing as well at this stage of experimentation. Becker^11^ and Kim^22^ also used color section extraction. Finally rotation was employed by Becker^11^, Kim^12^, Stidham^13^ and Ozawa^21^.

Figure 2 illustrates the methods used for feature extraction, including the VGG16 encoder proposed by Kim et al.^12^, InceptionV3 selected by Huang^14^, and ResNet50 employed by Fan^23^. Maurício et al.^20^ explored several models to evaluate the capabilities of both CNNs and ViTs in their study. Although the study comprises six models each from the ViT and CNN categories, only the most significant ones are highlighted in this section. These models include ViT-S/16, ViT-S/32, and ViT-B/16 from the ViT series, alongside ResNet50, InceptionV3, and VGG16 from the CNN series. In contrast to the limited use of feature extraction methods, a wide variety of machine learning architectures and techniques were employed by the researchers to advance the UC imaging domain. For instance, Huang^14^ utilized k-nearest neighbors, support vector machines, and deep neural networks, combined with ensemble learning, for classification. Kim^22^ employed MobileNetV2, MobileNetV3Large, and Xception for their tasks.

**Fig. 3:**
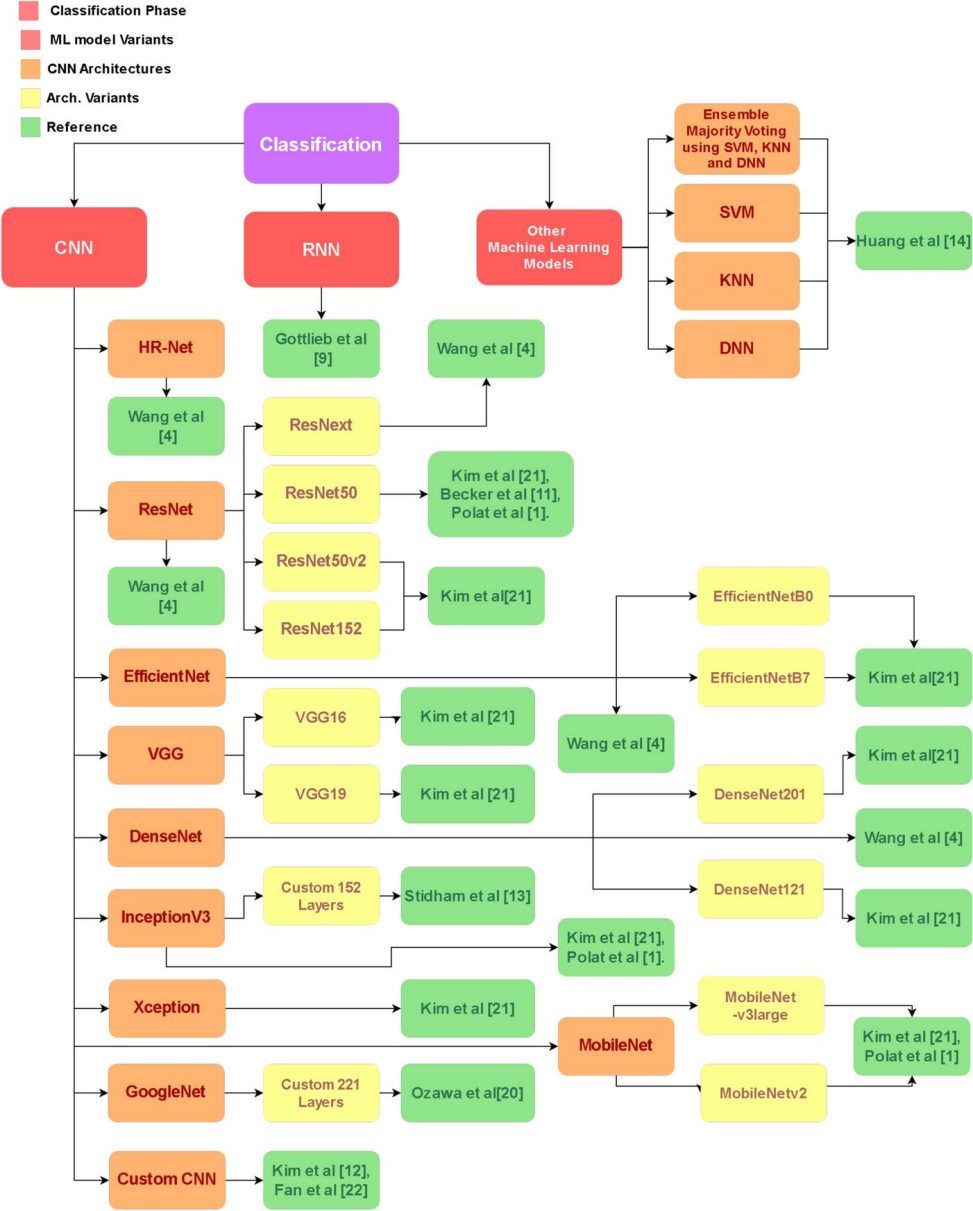
Classification methods proposed by researchers in recent years.

Figure 3 shows multiple methods for classification proposed by researchers in recent years. HRnet was used by Wang^4^ along with ResNeXt, while Quality Control and Ulcerative Colitis Scoring models were utilized by Becker^11^. EfficientNet was used by Wang et al.^4^ and Kim et al.^22^, while DenseNet was chosen by Wang et al.^4^, and Kim et al.^22^. ResNet family models were employed by Wang^4^, Becker et al.^11^, Kim et al.^22^, though Gottlieb et al.^9^ utilized Kim et al.^12,22^ used VGG16, while Fan et al.^23^ used a custom CNN for classification. GoogleNet was used by Ozawa et al.^21^ while VGG19 was employed by Kim et al.^22^. Finally InceptionV3 was utilized by Stidham^13^ and Kim^22^. After compilation and summarizing of the attained research material, It has been observed that in the scope of this study, several authors that have worked in this domain had undergone some common challenges that still hinder the progress of attainment of piratical resources for the actual development of useful Computer-Aided Diagnosis tools.

## Proposed methodology

**Fig. 4:**
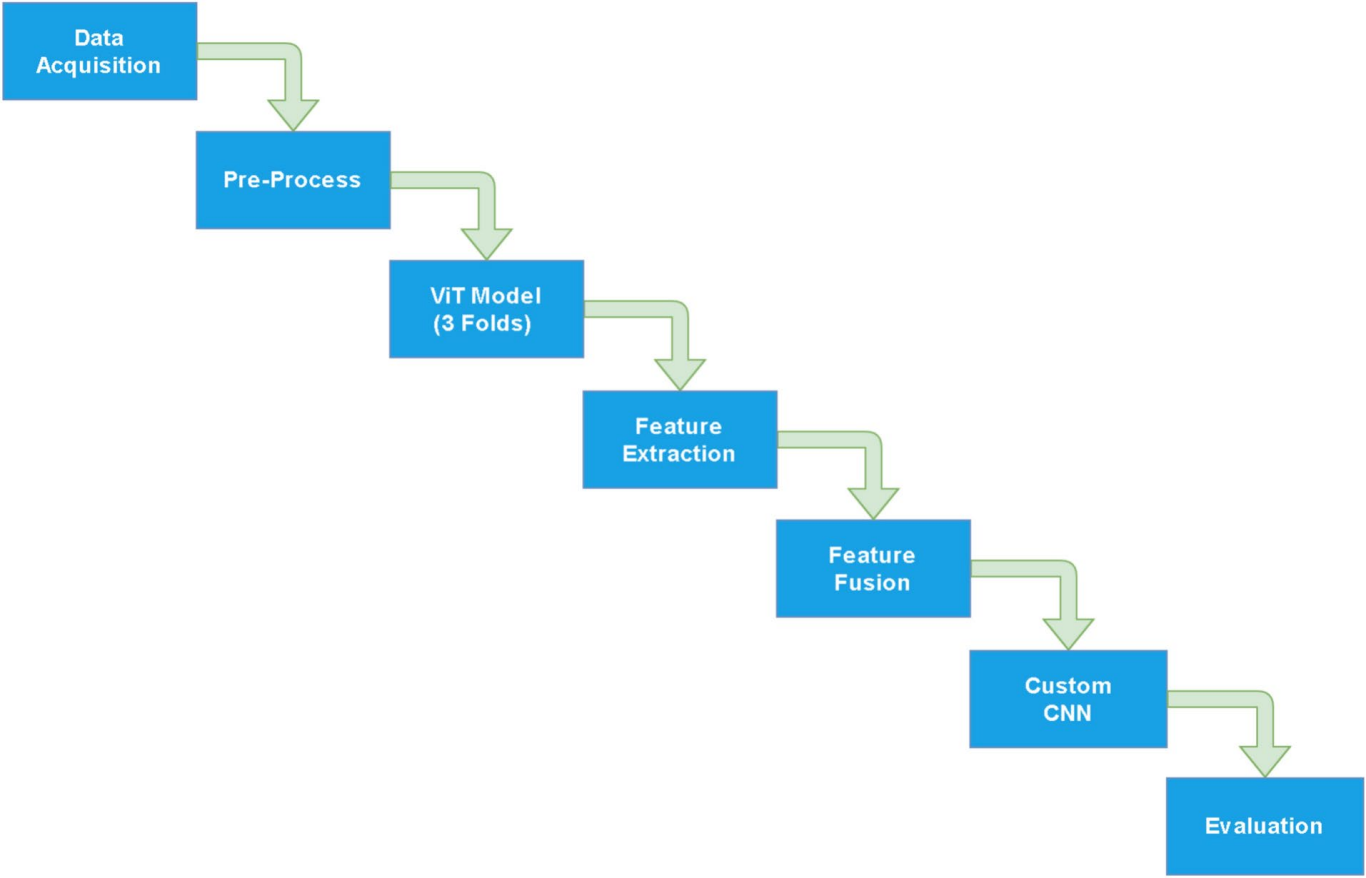
Figure illustrates overflow of the entire applied pipeline of the methodology.

The proposed methodology aims to address the challenges of class imbalance in Ulcerative Colitis endoscopic image datasets by utilizing a novel High-Frequency Balancing and Augmentation technique. Unlike traditional methods like oversampling or simple augmentation, this technique focuses on areas where minority classes are more concentrated, thereby avoiding over-representation and reducing the risk of overfitting. Oversampling, though commonly used, tends to amplify minority class samples across the entire dataset, leading to poor generalization^24^. Simple augmentation, on the other hand, applies transformations without specifically targeting minority classes, which may not effectively solve the imbalance problem^25^. Therefore, we avoided these techniques and opted for a method that better aligns with our objectives. The High-Frequency Balancing and Augmentation technique selectively balances the minority classes by introducing a dropout layer that ensures the retention of crucial patterns and features, preventing overfitting while addressing class imbalance. Figure 4 shows the flow diagram of the proposed method. It consists of data preprocessing, followed by feature extraction of a customized Vision Transformer (ViT) and then fusion of features and classification.

### Pre-processing

**Fig. 5:**
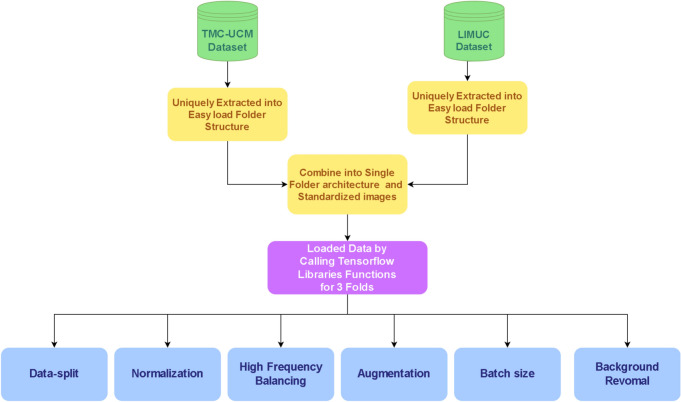
Proposed steps of preprocessing for UC detection.

The preprocessing steps are designed to standardize and optimize the input data, ensuring consistency across different datasets. First, data standardization is applied to address differences in scales or units among the datasets. This step ensures that the input data is uniform, leading to improved model performance. Figure 5 illustrates the preprocessing steps. The datasets used in this study, LIMUC^1^ and TMC-UCM, originally had resolutions of 352 $\times$ 288 and 300 $\times$ 300, respectively. These were standardized to 300 $\times$ 300 to maintain uniformity. Additionally, black filtering was applied to isolate key features, reduce noise, and enhance clarity in areas of interest. Normalization was also performed to mitigate fluctuations in pixel values from 0-255 to 0-1, ensuring that the data is well-prepared for model training. The preprocessing pipeline, including these steps, provides a solid foundation for the subsequent stages of the methodology.

To address the problem of class imbalance, we chose to avoid oversampling and simple augmentation. Oversampling tends to over-represent minority classes across the entire dataset, leading to poor generalization and potential overfitting. Simple augmentation applies transformations such as perspective, orientation, color, scale, brightness, and contrast without specifically targeting minority classes. Therefore, we implemented the High-Frequency Balancing and Augmentation technique, which focuses on areas where minority classes are more concentrated. This method not only balances the class distribution but also reduces overfitting by introducing a dropout layer that preserves crucial patterns and features. After applying this technique, the dataset comprises an average of 9103 images per class label (Table 1 provide detail info. regarding dataset pre and post the process phase) where dataset is split into a 80:10:10 ratio for training, validation and testing.

### Feature extraction

#### Modified ViT architecture

In the proposed methodology, we have made several key modifications to the standard Vision Transformer (ViT) architecture to enhance its performance on the Ulcerative Colitis endoscopic image dataset. These modifications are designed to optimize feature extraction, reduce overfitting, and better handle the class imbalance inherent in the dataset. The specific modifications are as follows:

- Patch size: The standard ViT typically uses larger patch sizes (e.g., 16 $\times$ 16 pixels). In our method, we reduced the patch size to 10 $\times$ 10 pixels. This modification allows the model to capture finer details in the endoscopic images, which are crucial for differentiating between different severity levels of ulcerative colitis.
- Attention heads: In the standard ViT, the number of attention heads can vary, but larger models often use more heads (e.g., 12 or 16). We have adjusted this to 4 attention heads, balancing computational efficiency with the model’s ability to capture relevant patterns in the data. This adjustment helps the model focus on important features without being overwhelmed by the complexity of the attention mechanism.
- Dropout layer: To mitigate overfitting, particularly given the class imbalance in the dataset, we introduced a dropout layer with a dropout rate of 0.1 after the transformer encoder. This addition helps the model generalize better by preventing it from relying too heavily on any specific features during training.
- MLP head customization: The Multi-Layer Perceptron (MLP) head of the ViT model has been fine-tuned to better handle the classification task specific to this dataset. While the standard ViT uses a generic MLP head, we optimized the hidden dimension to 768 and the MLP dimension to 3072, tailoring it to the unique characteristics of the ulcerative colitis dataset.

**Fig. 6:**
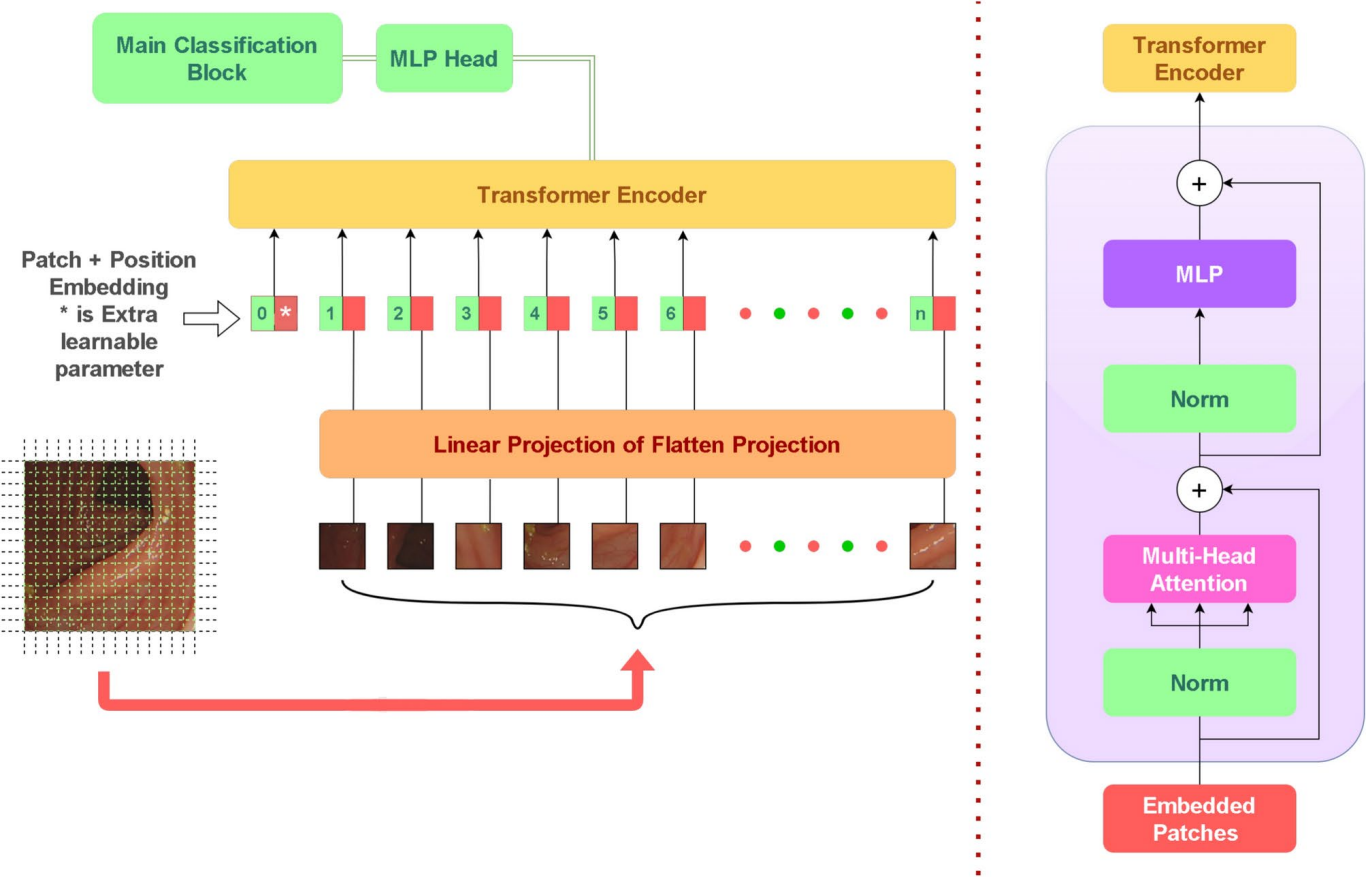
Proposed architecture of ViT for feature extraction.

**Fig. 7:**
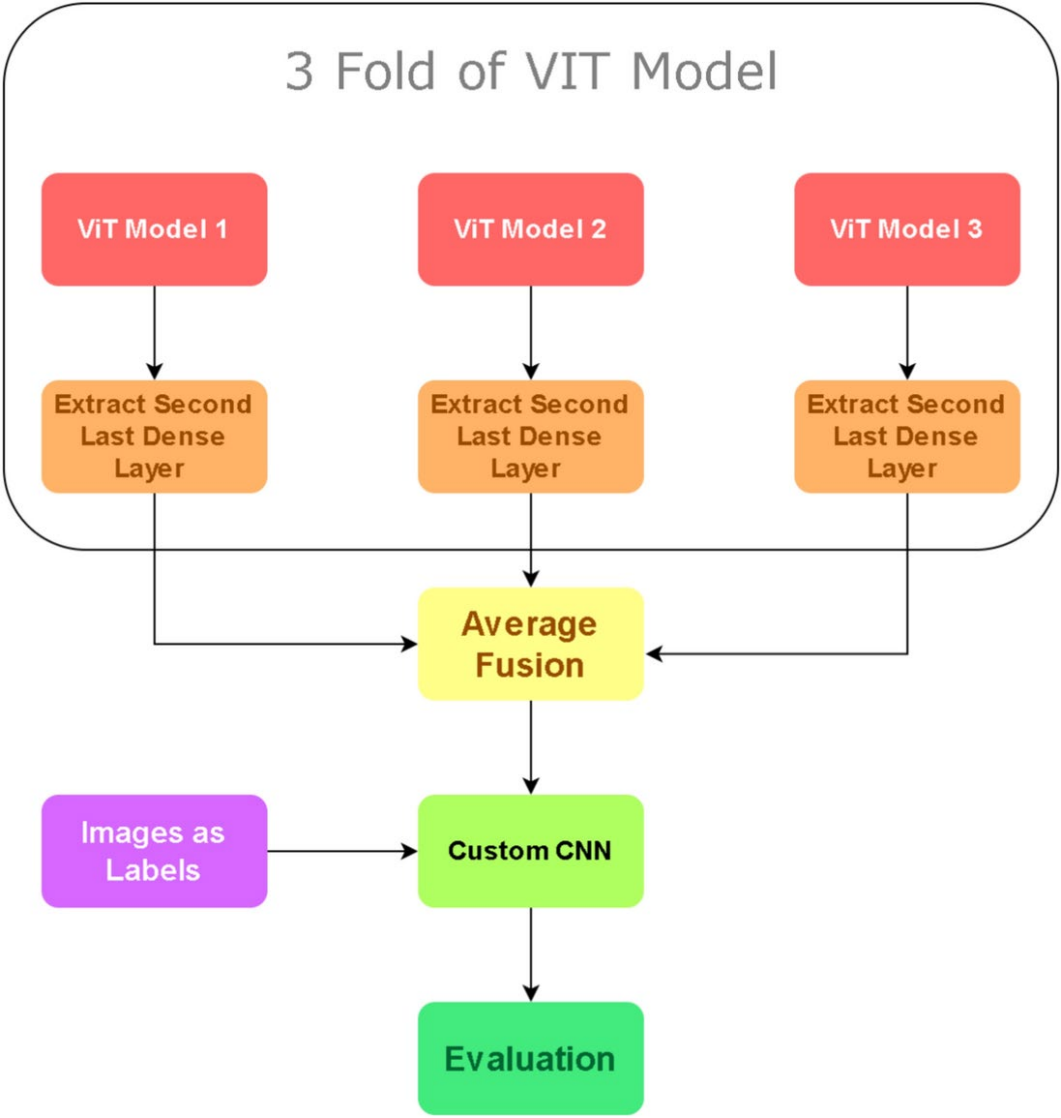
Averaging fusion on the ViT folds.

#### ViT architecture

However to understand how the Modified Vision Transformer infrastructure we need a general understanding of (ViT) processes image data compared to Convolutional Neural Networks (CNNs), thus requiring a look at their internal mechanisms. Figure 6 shows the architecture of the proposed ViT.Particularly the initial ability of ViTs to capture context.This approach ensures a holistic analysis of the different features in different regions of the dataset. The Vision Transformer architecture consists of a Patch extractor, Token Embedder, transformer encoder, Self-attention mechanism, and MLP head^26^. The Patch Extractor divides images into patches, which are then transformed into a lower-dimensional vector space using a linear transformation^27^. This process can be mathematically expressed as:

1 $$\begin{aligned} {\textbf{x}}_p = {\textbf{W}}_p \cdot {\textbf{x}} + {\textbf{b}}_p \end{aligned}$$

where, ${\textbf{x}}_p$ is the vector representation of the image patch, ${\textbf{W}}_p$ is the weight matrix for the linear transformation, ${\textbf{x}}$ is the original image patch and ${\textbf{b}}_p$ is the bias term. The Token Embedder embeds a token within the sequence of patches to represent the entire image. This embedding process can be described by^28^:

2 $$\begin{aligned} {\textbf{z}}_0 = \left[ {\textbf{x}}_{class}; {\textbf{x}}_p^1; {\textbf{x}}_p^2; \ldots ; {\textbf{x}}_p^N \right] + {\textbf{E}}_{pos} \end{aligned}$$

where, ${\textbf{z}}_0$ is the initial embedded sequence, ${\textbf{x}}_{class}$ is the class token, ${\textbf{x}}_p^i$ are the patch vectors and ${\textbf{E}}_{pos}$ is the positional encoding. The Transformer Encoder consists of the Self-Attention Mechanism and the Feed Forward Neural Network. The Self-Attention Mechanism points out the dependencies among patches and annotates their significance:

3 $$\begin{aligned} \text {Attention}({\textbf{Q}}, {\textbf{K}}, {\textbf{V}}) = \text {softmax}\left( \frac{{\textbf{Q}} {\textbf{K}}^\top }{\sqrt{d_k}} \right) {\textbf{V}} \end{aligned}$$

where, ${\textbf{Q}}$, ${\textbf{K}}$, and ${\textbf{V}}$ are the query, key, and value matrices and $d_k$ is the dimension of the key vectors^29^. The Feed Forward Neural Network captures complex relations within each patch:

4 $$\begin{aligned} \text {FFN}({\textbf{z}}) = \max \left( 0, {\textbf{z}} {\textbf{W}}_1 + {\textbf{b}}_1 \right) {\textbf{W}}_2 + {\textbf{b}}_2 \end{aligned}$$

where, ${\textbf{z}}$ is the input from the self-attention layer, ${\textbf{W}}_1$ and ${\textbf{W}}_2$ are weight matrices, ${\textbf{b}}_1$ and ${\textbf{b}}_2$ are bias terms. Finally, the MLP head undertakes classification and object detection:

5 $$\begin{aligned} {\hat{y}} = \text {MLP}({\textbf{z}}_L) \end{aligned}$$

where, ${\hat{y}}$ is the output prediction and ${\textbf{z}}_L$ is the output of the final transformer encoder layer. The MLP processes the patches through a series of connected layers containing non-linear activation functions like ReLU to perform higher classification and feature extraction^30,31^. For the intention of improving retrace—ability of the experiment the applied setting for the employed ViT model have been summarized as implication of Patch Size of 10 $\times$ 10 pixels, Number of Layers: 12, Hidden Dimension: 768, MLP Dimension: 3072, Number of Heads: 4, Dropout Rate: 0.1, Batch Size: 32, Learning Rate (LR): 1e-4 and 50 epochs are set for the applied experiment.

Feature extraction is performed using the hidden layers of a ViT model, which is configured with 768 hidden parameters. Now with results from the 3-fold ViT acquired, we extract the penultimate layer of each ViT fold to acquire aggregated features^28,32^. These 3 different feature vectors are then fused through the usage of the averaging fusion technique because fusion allows the unification of multiple representations into a single feature map, along with images of the combined datasets are fed to improve generalization^33,34^, Custom CNN is applied to further refine and enhance the feature vector obtained from the ViT. Initially, feature extraction is carried out using the penultimate layers of the 3-fold ViT, capturing complex patterns and contextual information. The extracted features are then passed through a Custom CNN to capture local patterns and enhance the feature representation further. This combination leverages both the global context captured by ViT and the detailed spatial features captured by CNN^33,34^.

**Fig. 8:**
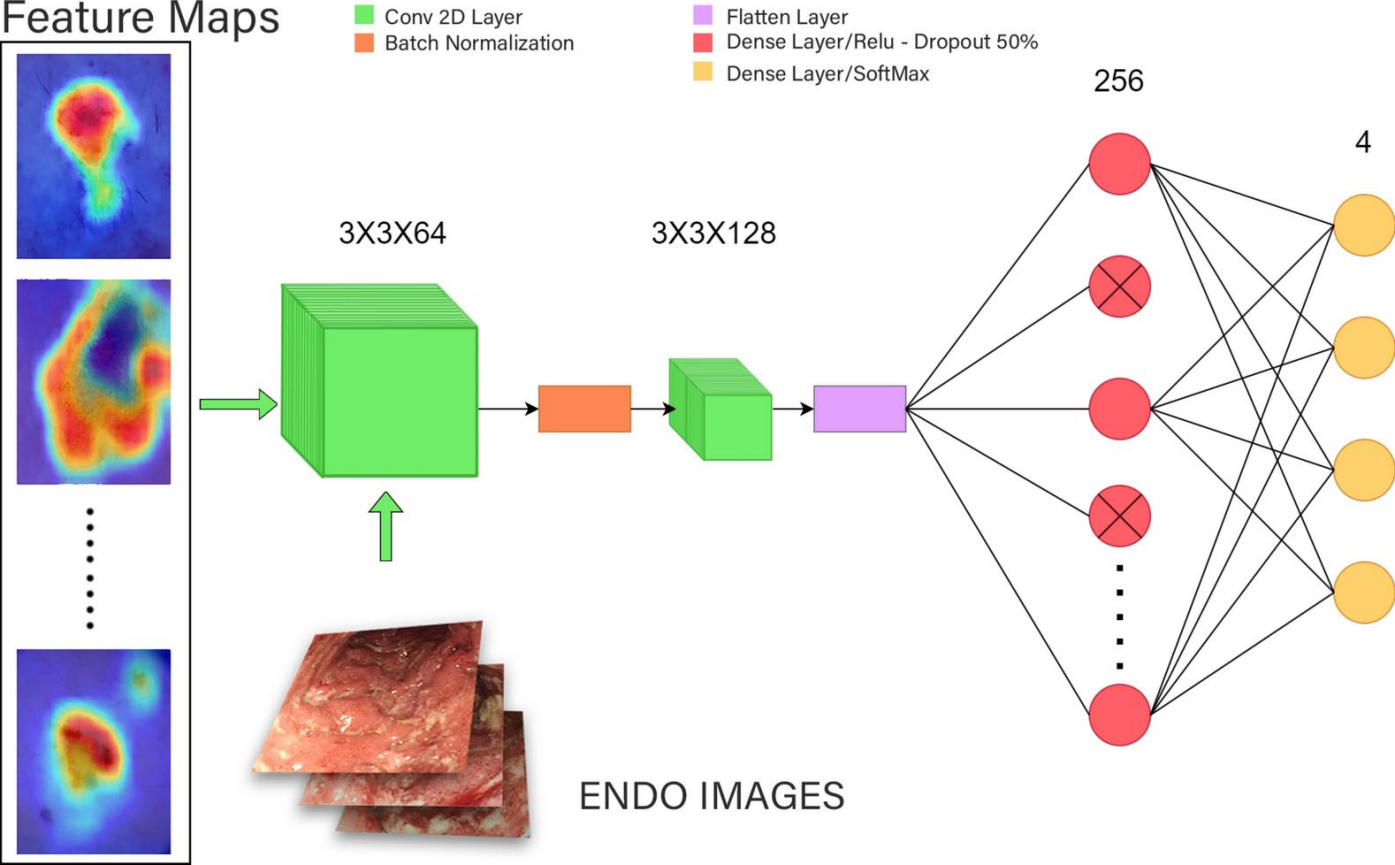
Architecture of custom CNN with feature fusion.

#### Classification

Feature vector is fed into a custom CNN architecture. The first layer is a Conv2D layer where kernels convolve across the dataset to create feature maps. This layer focuses on the detection of local patterns and spatial relationships while employing the activation function of ReLU to introduce non-linearity and diminish the impact of irrelevant features and the vanishing gradient, so that the model can learn higher-level features. Mathematically, the convolution operation can be expressed as:

6 $$\begin{aligned} \text {Conv2D}({\textbf{x}}) = \text {ReLU} \left( {\textbf{W}}_c *{\textbf{x}} + {\textbf{b}}_c \right) \end{aligned}$$

where, ${\textbf{W}}_c$ are the convolutional kernels, ${\textbf{x}}$ is the input feature map, ${\textbf{b}}_c$ is the bias term, $*$ denotes the convolution operation. The Batch Normalization technique optimizes the stability and speed of the network so that deeper layers can be utilized. It works by increasing the convergence by reducing the mean and variance of each layer while utilizing parameters like shift and scale that allow adaptation to specific data features. This can be expressed as:

7 $$\begin{aligned} \text {BN}({\textbf{x}}) = \gamma \left( \frac{{\textbf{x}} - \mu }{\sqrt{\sigma ^2 + \epsilon }} \right) + \beta \end{aligned}$$

where, $\mu$ and $\sigma ^2$ are the mean and variance of the input, $\gamma$ and $\beta$ are the learnable scale and shift parameters and $\epsilon$ is a small constant for numerical stability. The Flatten layer reduces the dimensionality of the input to linearity through concatenation, which allows focusing of all dimensions onto a single axis. This is typically used when transitioning from convolutional layers to fully connected layers. It can be described as:

8 $$\begin{aligned} \text {Flatten}({\textbf{x}}) = \text {reshape}({\textbf{x}}, [-1]) \end{aligned}$$

where the input ${\textbf{x}}$ is reshaped into a single-dimensional vector. Figure 7 shows the proposed method for fusion of ViTs, whereas, Figure 8 shows the proposed feature fusion of CNNs. The fully connected layer, referred to as the Dense Layer, performs a linear operation followed by a non-linear activation function such as ReLU, GeLU, tanh, etc. This layer performs higher-level feature extraction and classification by utilizing all the information from previous layers to make predictions. The operation can be described as:

9 $$\begin{aligned} \text {Dense}({\textbf{x}}) = \sigma ({\textbf{W}}_d {\textbf{x}} + {\textbf{b}}_d) \end{aligned}$$

where, ${\textbf{W}}_d$ is the weight matrix, ${\textbf{x}}$ is the input vector, ${\textbf{b}}_d$ is the bias term and $\sigma$ is the activation function. Finally, a regularization technique referred to as the Dropout Layer is utilized to prevent over-fitting. This layer introduces redundancy into the network through random deactivation of a small fraction of the total neurons in a layer during each fold. This allows the model to be better generalized by reducing its dependency on certain neurons for analysis, thus forcing the model to avoid memorizing the data too closely. The number of neurons to be deactivated each time is completely determinable by the user. The dropout operation can be represented as:

10 $$\begin{aligned} \text {Dropout}({\textbf{x}}) = {\textbf{x}} \odot {\textbf{r}} \end{aligned}$$

where, $\odot$ denotes element-wise multiplication and ${\textbf{r}}$ is a binary mask vector with a certain probability of zero entries. To put it shortly the architecture of CNN model used in feature fused CNN model comprises of 2 Conv2D layer where the first layer uses 64 kernels, of size 3*3 and second uses 128 kernels of the same 3*3 size after which a flatten layer is applied with 2 Dense Layers of 256 density with activation function of relu and and 4 Density to specify class labels with softmax as activation function. In between the Dense Layers a Dropout of 0.5 is applied to ensure optimized training of neuron in each section of applied Dense Layers.

## Datasets

The two datasets LIMUC^1^ and TMC-UCM have been used in this research.

### LIMUC dataset

Labelled Images for Ulcerative Colitis^1^ (LIMUC) dataset was compiled by the Marmara University School of Medicine’s Department of Gastroenterology using 11,276 Ulcerative Colitis images of size 352 $\times$ 288. Having acquired these diagnoses from 564 patients through 1043 colonoscopy exams carried out from December 20111 to July 2019, the dataset was assessed by two gastroenterology experts who classified using the Mayo Endoscopic Scoring system. Then, a third-party professional independently assessed the data and assigned Mayo scores without any knowledge of the prior assessment, with the final Mayo score being assigned through a majority vote system.

### TMC-UCM dataset

Originating from the Tongji Medical College of Huazhong University of Science and Technology in Wuhan, China, this publicly available dataset is derived from the tests performed on 308 patients with Ulcerative Colitis^4^, who underwent colonoscopy based on IBD guideline between January 2014 and December 2021. It contains 12,163 images obtained through the Olympus colonoscopy, which, when filtered to exclude images with stool, blur, or halation, is reduced to 7978 images. This dataset was reviewed by at least three expert gastroenterologists with sufficient experience in tackling IBDs, who resolved any judgmental differences through discussion, and even then, their final classification decision on the basis of MES was further reviewed by another IBD specialist.

**Fig. 9:**
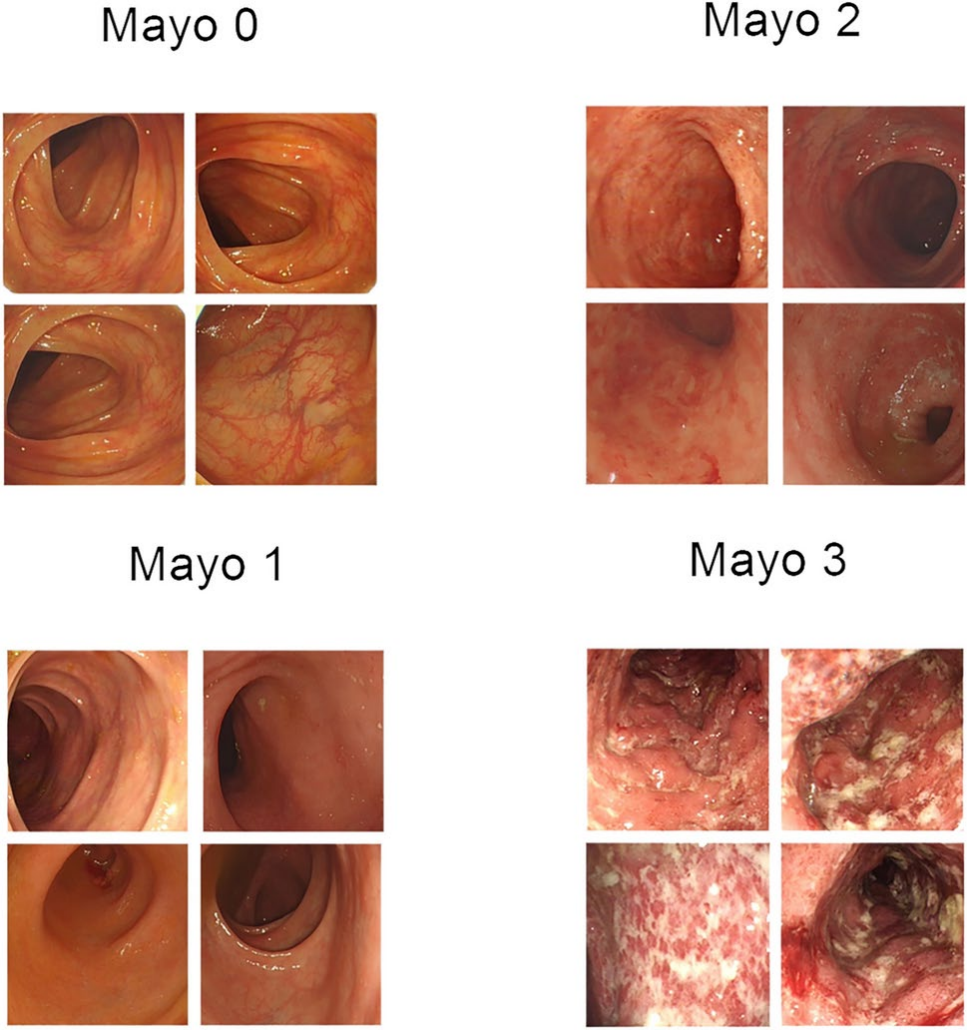
Figure illustrates the visual aspects of the applied images.

Associated with the diagnosis of Ulcerative Colitis are the different diagnosis systems used to identify the stage of progression of the disease, such as UCEIS and MES. In our research, we utilize the Mayo Endoscopic Scoring system, which assigns four categories, ranging from 0 to 3, to the different disease progression stages. Mayo 0 indicates no inflammation with the surface being smooth and healthy with a normal mucosa. Mayo 1 represents a mild stage of inflammation where the mucosa might be irregular or granular, though no erosions or ulcers can be found. Further signs can be mild erythema and friability. Mayo 2 symbolizes moderate inflammation, where limited ulceration is identifiable, along with more pronounced signs like erosion, friability, and marked erythema. The final Mayo 3 stage is potentially fatal due to severe inflammation marked by extensive ulcerations and spontaneous bleeding. Furthermore, the presence of a pseudo-polypoid is also possible. Table 1 presents the detailed description of datasets used in this study, whereas, Fig. 9 shows the sample images of the dataset.

**Table 1:** Description of datasets used in this research.

Dataset name	Total_images	Mayo0	Mayo1	Mayo2	Mayo3	Dimensions
LIMUC	11,276	6105	3052	1254	865	352*288
TMC-UCM	7978	3031	1835	1675	1423	300*300
After Pre-process	36,413	9104	9103	9103	9103	300*300

## Results

We have trained and tested the proposed method on the two datasets. To address the validation distribution across 10 folds during the cross-validation process. Each fold represents a different subset of the data, used to evaluate the model’s performance. The consistency in validation accuracy across the folds indicates the model’s ability to generalize well to unseen data. Additionally, the relatively low standard deviation suggests that the model’s performance is stable and not overly sensitive to the specific data partitions. These results highlight the robustness of the model and its potential for reliable predictions in real-world applications. Where mean is calculated by applying given expression:

11 $$\begin{aligned} \mu = \frac{1}{n} \sum _{i=1}^{n} x_i \end{aligned}$$

Similarly Standard deviation is expressed as:

12 $$\begin{aligned} \sigma = \sqrt{\frac{1}{n} \sum _{i=1}^{n} (x_i - \mu )^2} \end{aligned}$$

In this research, we have used performance measures including AUC-ROC, precision, F-1 score and recall to validate the performance of the proposed method. ROC refers to the Receiver Operating Characteristic, which is a graphical plot drawn on the basis of the discriminating thresholds being varied so that the trade-offs between sensitivity and precision can be displayed. It is derived through the calculation of specificity and sensitivity.

13 $$\begin{aligned} \text {ROC} = \{ (\text {Specificity}_i, \text {Precision}_i) \}_{i=0}^n \end{aligned}$$

Specificity measures the proportion of true negatives that are correctly identified by the neural network architecture. It is calculated through the following formula:

14 $$\begin{aligned} \text {Specificity} = \frac{\text {TN}}{\text {FP} + \text {TN}} \end{aligned}$$

Where:

- FP = False Positives
- TN = True Negatives

Precision refers to the proportion of true positives that are correctly identified by the neural network. Its calculation is carried out through the following process:

15 $$\begin{aligned} \text {Sensitivity} = \frac{\text {TP}}{\text {FP} + \text {TP}} \end{aligned}$$

Where:

- TP = True Positives
- FP = False Positives

AUC refers to Area Under the ROC Curve, which is a singular value between 0 and 1 that represents the overall efficiency of the classifier.

16 $$\begin{aligned} \text {AUC} = \int _{0}^{n} \text {Sensitivity}(\text {Specificity}) \, d\text {Specificity} \end{aligned}$$

There are multiple reasons for not using accuracy. Firstly, medical datasets can suffer from class imbalance due to some conditions being rarer than others, thus generating a difference in the weight of different severity classes. Therefore, a model achieving high accuracy could simply be doing so through bias towards the majority class. Moreover, accuracy is unable to account for the different types of errors i.e., false positives and false negatives in imbalance datasets. In contrast, AUC-ROC addresses the class imbalance by considering all possible thresholds of classification, which leads to it being less affected by any imbalance if present, and therefore provides for a more robust measurement of effectiveness. It also captures two critical aspects of diagnosis through a single measure: true positives and true negatives or sensitivity and precision. This means that AUC-ROC can declare both a diseased state when there is a disease and a non-diseased state when there is none. In the medical field, where false positives and false negatives can constitute serious consequences, AUC-ROC is a better measure due to balancing these considerations of clinical requirements or the costs associated with diagnostic errors through adjustment to the thresholds of classification probability. The ROC curve provides a comprehensive overview regarding the trade-offs involved in the binary classification methods by plotting the True Positive rate against the False Positive rate.

Recall is a metric of measure which involves the identification of correct samples are identified from the overall dataset by the performing model under study it is described by the following formula:

17 $$\begin{aligned} R = \frac{TP}{TP + FN} \end{aligned}$$

F-1 score another metric of measure used in machine learning It serves as a harmonic mean between precision and recall and is described by the following formula:

18 $$\begin{aligned} F1 = 2 \cdot \frac{Precision \cdot Recall}{Precision + Recall} \end{aligned}$$

**Table 2:** Comparative analysis of results obtained through ablation stud performed by varying experimental settings.

Model	AUC-ROC	Precision				F1-Score				Recall			
		M0	M1	M2	M3	M0	M1	M2	M3	M0	M1	M2	M3
Standardized dataset VGG-16 Model	0.54	0.62	0.48	0.41	0.44	0.52	0.24	0.37	0.58	0.45	0.22	0.33	0.55
Standardized dataset VGG-22 Model	0.56	0.64	0.52	0.68	0.47	0.49	0.21	0.5	0.53	0.41	0.19	0.47	0.62
Standardized dataset Res-Net 50 Model	0.51	0.55	0.39	0.4	0.42	0.47	0.31	0.39	0.41	0.35	0.3	0.37	0.4
Standardized dataset ResNet 101 model	0.5	0.51	0.35	0.43	0.41	0.48	0.28	0.4	0.43	0.34	0.25	0.38	0.42
Standardized dataset Modified ViT model	0.8	0.8	0.71	0.82	0.77	0.78	0.65	0.79	0.8	0.75	0.62	0.77	0.79
Standardized datastet CustomCNN model	0.85	0.83	0.67	0.84	0.79	0.81	0.75	0.82	0.8	0.77	0.68	0.8	0.78
Standardized dataset + High frequency balancing VGG-16 model	0.5865	0.665	0.5	0.43	0.46	0.54	0.25	0.415	0.58	0.47	0.23	0.38	0.645
Standardized dataset + High frequency balancing VGG-22 model	0.599	0.685	0.575	0.72	0.49	0.535	0.25	0.5	0.585	0.46	0.23	0.48	0.685
Standardized dataset + High frequency Balancing ResNet 50 model	0.5282	0.58	0.43	0.45	0.46	0.53	0.355	0.44	0.455	0.41	0.33	0.43	0.45
Standardized dataset + High frequency balancing ResNet 101 model	0.53	0.57	0.405	0.475	0.455	0.54	0.335	0.45	0.46	0.41	0.315	0.44	0.46
Standardized dataset + High frequency balancing Modified ViT model	0.845	0.855	0.73	0.885	0.83	0.845	0.71	0.87	0.845	0.82	0.75	0.84	0.845
Standardized dataset + High frequency balancing custom CNN model	0.895	0.87	0.72	0.895	0.84	0.85	0.815	0.865	0.84	0.84	0.725	0.875	0.835
Standardized dataset + High frequency balancing and augmentation VGG-16 model	0.593	0.67	0.50	0.43	0.46	0.54	0.25	0.42	0.58	0.47	0.23	0.39	0.70
Standardized dataset + High frequency balancing and augmentation VGG-22 model	0.599	0.69	0.59	0.72	0.49	0.54	0.25	0.50	0.60	0.47	0.23	0.48	0.71
Standardized dataset + High frequency balancing and augmentation Res-Net 50 Model	0.5282	0.58	0.43	0.46	0.46	0.55	0.36	0.45	0.46	0.43	0.33	0.45	0.46
Standardized dataset + High frequency balancing and augmentation ResNet 101 model	0.53	0.59	0.42	0.48	0.46	0.56	0.35	0.46	0.46	0.44	0.32	0.46	0.46
Standardized dataset + High frequency balancing and augmentation modified ViT model	0.85	0.87	0.73	0.91	0.85	0.87	0.73	0.91	0.85	0.85	0.84	0.87	0.86
Standardized dataset + High frequency balancing and augmentation CustomCNN model	0.90	0.87	0.73	0.91	0.85	0.85	0.84	0.87	0.86	0.87	0.73	0.91	0.85

### Experimental results

**Fig. 10:**
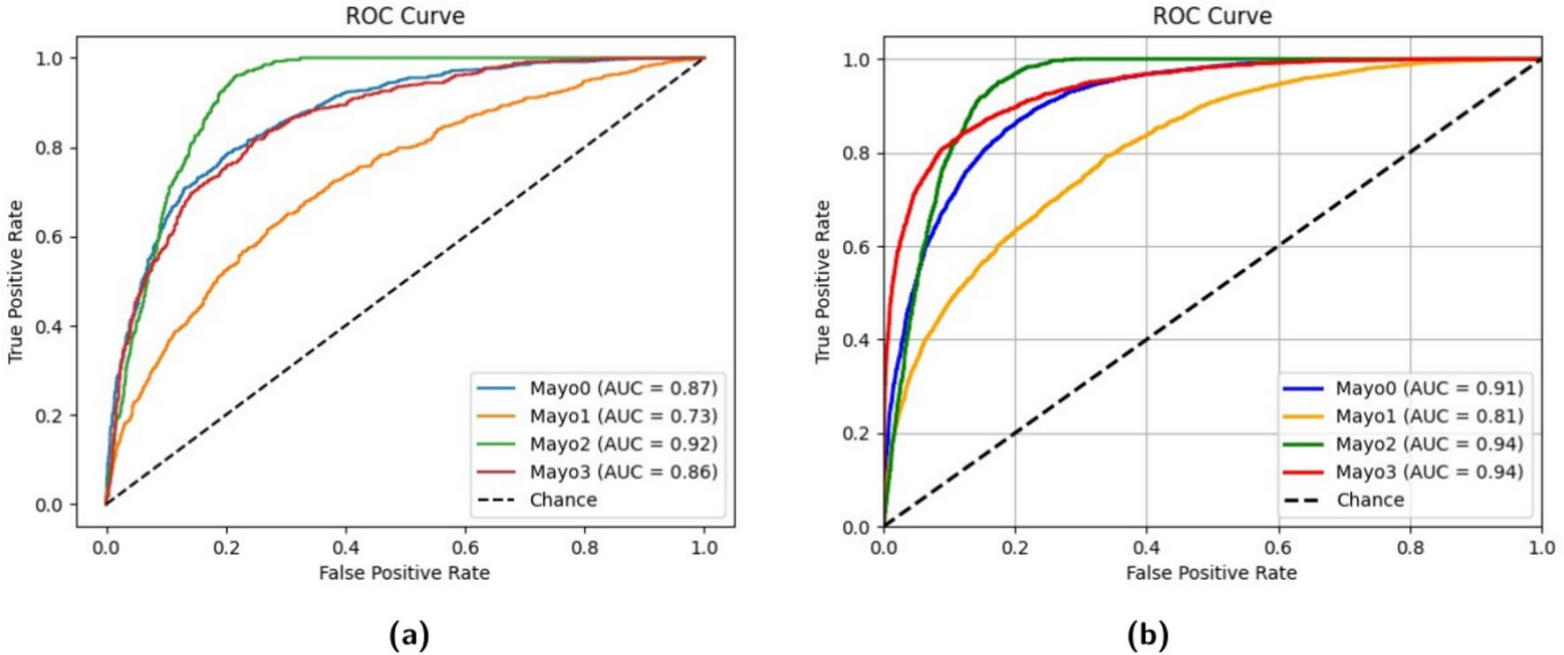
Compariosn of ROC curves for multiple classes using ViT and Custom CNN.

Table 2 compares the results achieved on different CNNs models with ViT and custom CNN. It has been observed from ablation study that the performance of the CNN increases the correction rate in all the Mayo classes significantly. Furthermore, the overall performance of the CNN model displays an increase from the ViT’s 85% to the CNN’s 90%. We used *k* fold cross validation method to split the data into train and test with value of *k* as 10 and achieved an average accuracy of 90.453% using standard deviation of 0.431. Figure 10 presents the ROC curves of ViT and Custom CNN and it shows that the scores for different classes of Ulcerative Colitis are attained by the best fold of the 3-fold ViT model are 0.87 (Mayo 0), 0.73 (Mayo 1), 0.92 (Mayo 2), and 0.86 (Mayo 3). In contrast to these results are those of the feature-fused CNN, which provided scores of 0.91, 0.81, 0.94, and 0.94 for the Mayo 0, 1, 2, and 3 categories, respectively. It is noticeable that the performance of the CNN increases the correction rate in all the Mayo classes significantly. Furthermore, the overall performance of the CNN model displays an increase from the ViT’s 85 to the CNN’s 90.

**Fig. 11:**
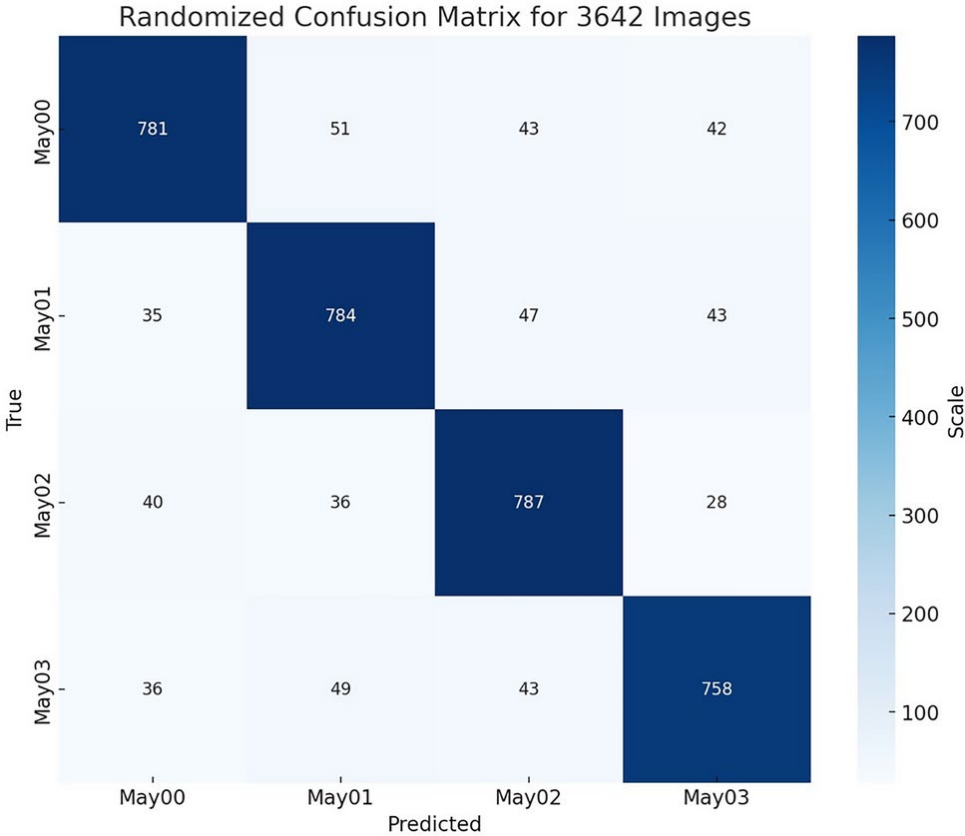
Confusion Matrix achieved by Custom CNN model.

Figure 11 presents the confusion matrix to show the performance of the Custom CNN model attained by averaging fusion and illustrates the True Positives, False Positives, True Negatives, and False Negative prediction of labels for each label class in the study. Table 3 compares the performance of various state-of-the-art models used in the classification of ulcerative colitis severity with our proposed model. The key metrics presented include the methodology, total parameters, and the performance in terms of accuracy and AUC-ROC scores.

**Table 3:** Comparison of results and complexity of the proposed method with existing state of the art methods.

Studies	Methodology	Number of parameters	Accuracy/AUC-ROC
Wang et al.^4^,	CB-HRnet	Lies somewhere (10.6–60.1) mill	Accuracy MES 0/1/2/3: 82.45%, MES 0/1/23: 87.37%, MES 0/123: 92.17% ,MES 01/23: 94.36%
Becker et al.^11^,	Q.C Model + UCModel(Both ResNet50)	25.6 million each	Accuracy0.84 $MES \ge 1$, 0.85 $MES \ge 2$, 0.85 $MES \ge 3$
Gottlieb et al.^9^,	CNN Visual Clarity and Bowel Prep Sore + RNN	–	Endoscopic Mayo AUC-ROC Score Mayo 0: 0.921, Mayo 1: 0.845, Mayo 2: 0.784, Mayo 3: 0.685.
Polat et al.^1^,	InceptionV3 Transfer learning model	23,851,784	Accuracy Mayo 0: 0.817, Mayo 1: 0.768, Mayo 2: 0.677, Mayo 3: 0.669.
Ozawa et al.^21^,	GoogLeNet (22layer)	9,888,8409,890,888 (as Composes on 2 Inception models in structure )	MES 0: AUC 0.86, MES 0-1: AUC 0.98.
Proposed study	Averaging Fusing 3 fold ViTs Trained + Custom CNN	18,427,140	AUC-ROC Mayo 0: 91%, Mayo 1: 81%, Mayo 2: 94%, Mayo 3: 94%.

While our comparison focuses on the critical performance metrics of accuracy and AUC-ROC, which are directly relevant to the evaluation of model effectiveness in the classification of ulcerative colitis, we acknowledge that certain complexity metrics (e.g., parameter count, FLOPs, inference time) are not available from the cited studies. These metrics are often underreported in the literature, limiting a direct comparison of computational complexity across models. However, based on the architectures employed, it can be inferred that models such as ResNet50 and GoogLeNet are generally more complex due to their deeper architectures and larger parameter counts compared to our custom CNN model. For instance, ResNet50 consists of 50 layers and GoogLeNet has 22 layers, both of which imply a higher computational cost and potentially longer inference times compared to our approach, which was designed with efficiency in mind.

Our custom CNN, combined with the High-Frequency Balancing and Augmentation technique, offers a streamlined architecture that may have advantages in terms of training and inference efficiency. This is particularly important given the targeted preprocessing steps we employed, which help reduce the need for extensive data augmentation and computational resources. These design choices likely contribute to the competitive performance observed in our AUC-ROC scores across the different class labels. We recognize the importance of providing a comprehensive evaluation of both performance and efficiency. Therefore, future work will aim to include more detailed complexity metrics, such as parameter count, inference time, and FLOPs, to better quantify the differences between our proposed method and existing state-of-the-art models. This will enable a more thorough assessment of the trade-offs between model complexity and performance.

The advent of Vision Transformers (ViTs) has introduced numerous advantages over Convolutional Neural Networks (CNNs), especially regarding size limitations and the ability to grasp overall features for accurate classification^32^. ViTs utilize self-attention mechanisms to directly capture global context, allowing them to uncover and link relationships between distant patches more effectively. This global perspective enables ViTs to generalize more efficiently on smaller datasets compared to CNNs^30^, which depend on local feature extraction and often require extensive data augmentation and large datasets for similar performance. Furthermore, ViTs can leverage pre-training on large, diverse datasets and fine-tuning on smaller, task-specific datasets, enhancing their adaptability and efficiency in data-constrained scenarios.

Through our experimentation, we uncovered that even if we utilized strategies that had been established by previous research, such as attaining optimized overall accuracy and AUC, the CNN models still consistently displayed poor results. Not only the overall accuracy but also the AUC metric also remained disappointingly low for the various architectures, leading to not-so-satisfactory results being obtained through their performance. Amidst these impediments that threatened to detail the course of experimentation, our research attempts with the Vision Transformer showed promising results through its 85% accuracy and a high AUC-ROC score. Though the exact factors that obstructed the performance of the failed CNN models require further investigation, we can discern that the unique characteristics of the ViT architecture must have played a crucial role in bypassing these impediment factors. Therefore, for greater accuracy, reliability, and robustness, ViT must be utilized in the future due to it possibly being the key to uncovering crucial insights into UC that could contribute greatly to speedy diagnosis and planning targeted recovery plans.

It has been concluded from the comparison of results obtained with state of the art existing methods that the proposed method is practical, realistic, and robust tool to medical practitioners for the correct multistage classification of Ulcerative Colitis severity on the basis of the Mayo scoring system, from 0 to 3, through the usage of endoscopic data. Using state-of-the-art architectures like Vision Transformer (ViT) classifier and custom Convolutional Neural Network (CNN) feature fusion, promising results have been acquired, as evidenced by the high accuracy in efficient categorization of disease severity. For practicality, as uncovered from Becker Paper implication of noisy labels helps optimize the performance of the model for practical situations, however a key limitation of the study was the limitation of the bias of the introduced labels as of implication of automatics means but with the availability of LIMUC dataset upon inspection several clearly identified labels had an amount of noise inside the images due presence of assisting diagnosis tools in the images using these images could help the performance of the model where the clearer images in quality of disease could highlight light common key features to capture from images and promote for more practical classification capability or model robustness. However, the shortcoming of our research is the lack of external validity, which is a crucial component to prepare for the deployment of machine learning models into real-world scenarios. External Validation works by testing the model on hitherto unknown data so as to gain an idea of the generalization capabilities of the model to assess its robustness.

## Conclusion

Our methodology of utilizing a 3-fold Vision Transformer (ViT) on the LIMUC and TMC-UCM datasets, which are optimized for usage through the implementation of High-frequency Balancing and Augmentation strategy to address any class imbalance problems, allows us to acquire three different sets of learned parameters which are then combined using averaging fusion into a single feature map of considerable generalization that is afterward used as the input along with the acquired dataset into the CNN, which yields improved results in terms of classification efficiency. The ViT model by itself provides an accuracy of 85% with the corresponding scores being Mayo0 (0.8828), Mayo1 (0.7431), Mayo2 (0.9327), and Mayo3 (0.8847), but the usage of the feature map along with the dataset for the CNN provides 90% accuracy with the scores of the four categories being Mayo0 (0.91), Mayo1 (0.81), Mayo2 (0.94), and Mayo3 (0.94). which have been applied to the attained open source datasets (as described above) by the implementation of optimal data balance and pre-processing. In the future, a multimodal approach could also prove productive for a superior understanding of UC and may help discover novel biomarkers that could contribute to a better therapeutic response. We can also deploy our developed model in real-world medical settings so that validation studies under the supervision of gastroenterologists and endoscopists can be carried out.

## Data Availability

The datasets analysed during the current study are available in the LIMUC and TMC-UCM repository. LIMUC can be directly accessed with the help of following first link. To access the TMC-UCM dataset, it is necessary to create a Baidu account and use it to retrieve the files. Baidu imposes certain regional restrictions, which may require users to have a local (mainland Chinese) phone number in order to register and access the data using the second link. https://zenodo.org/records/5827695#.Yi8GJ3pByUk;https://pan.baidu.com/share/init?surl=Q09eWJAQgkZf4lrvE5 hkKg &pwd=HUST. fuhao_zou@hust.edu.cn and yuqin@tjh.tjmu.edu.cn can also be contacted to get this TMC-UCM dataset.
